# The Role of Artificial Intelligence in Urogynecology: Current Applications and Future Prospects

**DOI:** 10.3390/diagnostics15030274

**Published:** 2025-01-24

**Authors:** Maria Beatriz Macedo de Oliveira, Francisco Mendes, Miguel Martins, Pedro Cardoso, João Fonseca, Teresa Mascarenhas, Miguel Mascarenhas Saraiva

**Affiliations:** 1 Faculty of Medicine, University of Porto, Alameda Professor Hernâni Monteiro, 4200-427 Porto, Portugal; beamoliveira14@gmail.com (M.B.M.d.O.); pedromarilio@gmail.com (P.C.); tqc@sapo.pt (T.M.); 2 Department of Gastroenterology, São João University Hospital, Alameda Professor Hernâni Monteiro, 4200-427 Porto, Portugal; francisco.cnm@gmail.com (F.M.); miguelpedro96@gmail.com (M.M.); 3 WGO Gastroenterology and Hepatology Training Center, 4200-427 Porto, Portugal; 4 CINTESIS@RISE, Department of Community Medicine, Information and Health Decision Sciences (MEDCIDS), Faculty of Medicine, University of Porto, 4200-427 Porto, Portugal; fonseca.ja@gmail.com; 5 Department of Obstetrics and Gynecology, São João University Hospital, Alameda Professor Hernâni Monteiro, 4200-427 Porto, Portugal

**Keywords:** artificial intelligence, gynecology, urogynecology, machine learning, deep learning

## Abstract

Artificial intelligence (AI) is the new medical hot topic, being applied mainly in specialties with a strong imaging component. In the domain of gynecology, AI has been tested and shown vast potential in several areas with promising results, with an emphasis on oncology. However, fewer studies have been made focusing on urogynecology, a branch of gynecology known for using multiple imaging exams (IEs) and tests in the management of women’s pelvic floor health. This review aims to illustrate the current state of AI in urogynecology, namely with the use of machine learning (ML) and deep learning (DL) in diagnostics and as imaging tools, discuss possible future prospects for AI in this field, and go over its limitations that challenge its safe implementation.

## 1. Introduction

### 1.1. Definition and Scope

Artificial intelligence (AI) is a type of computer system that mimics our brains’ intelligence and capacity to learn, process information, solve problems, and establish critical thinking to perform tasks, and whose growth and widespread has been remarkable in many areas, including medicine [1,2,3].

Healthcare-related AI has three categories: patient, clinician, and administrative-oriented, and has significant applications in diagnosis, prognosis, drug discovery, and development, improving communication, matching symptoms to appropriate physicians, transcribing medical documents, organizing and classifying images and remote treatment, thus reducing physicians’ workload, healthcare costs and improving patient outcomes [4,5]. Some AI applications in medicine include ECG interpretation, lung nodule detection on X-rays, and differentiation of high and low-grade dysplasia in colposcopy [6,7].

Urogynecology focuses on pelvic floor disorders (PFDs), including pelvic organ prolapse (POP), urinary incontinence (UI), and urinary tract infections (UTIs). AI enhances physicians’ clinical practice by assisting in diagnosis, tailored treatment and surgical planning well-informed decision-making, improving diagnostic accuracy, mitigating misdiagnosis, and offering personalized care. AI allows patient monitoring from afar or during the post-operative period through wearable devices (WDs) and sensors, enabling early interventions while reducing the probability of complications and hospital readmissions [8]. Urogynecology has a strong imaging component, using ultrasound, magnetic resonance imaging (MRI), and computer tomography (CT), which enables AI to be applied in image recognition, processing, reconstruction, automated analysis, and classification [3,9].

### 1.2. Historical Perspective

AI emerged in the 20th century, appearing in works like Turing’s test, and going through three waves of research and development since the 1950s. Currently, tremendous progress is being made and accelerated by deep learning (DL), responsible for AI’s third wave, contributing to its growing popularity and incorporation into our daily lives [10,11].

Over the years, there has been an increase in healthcare data availability and rapid development of big data analytic methods, which favored AI’s use in healthcare like machine learning (ML), a domain used for analyzing imaging exams (IEs), genetic data, laboratory exams and predicting the probability of certain outcomes, giving healthcare a computational decision-making tool to help physicians, opening the door for precision medicine [12,13,14].

### 1.3. Objectives of the Review

While AI is positively impacting several medical specialties, its application in urogynecology is recent and in its initial phases. Consequently, AI literature is still scarce on urogynecology. This review aims to illustrate the current state of AI in urogynecology, discuss ML and DL advances that benefit this area, analyze its limitations, and explore future prospects.

## 2. Chapter 1: AI Fundamentals and Techniques

### 2.1. Basic Concepts of AI

AI combines computer science and robust datasets, allowing problem-solving like humans do [15]. AI has a significant presence in our daily lives, being found on search engines, recommendation systems, facial recognition systems in smartphones, or in entities like ChatGPT powered by GPT-4o (OpenAI^TM^, San Francisco, CA, USA) [3,16,17].

To comprehend this domain, it is important to acknowledge that AI, ML, and DL are overlapping disciplines [18]. ML is a subset of AI that focuses on how computers learn with data, recognizing patterns and processing raw input data, employing a computer analysis that enables predictions of output values within an acceptable accuracy interval [6,19,20,21]. It can identify patterns and trends and learn through past experiences [21]. Thus, the algorithm can learn new tasks without being programmed for them.

**Figure 1:**
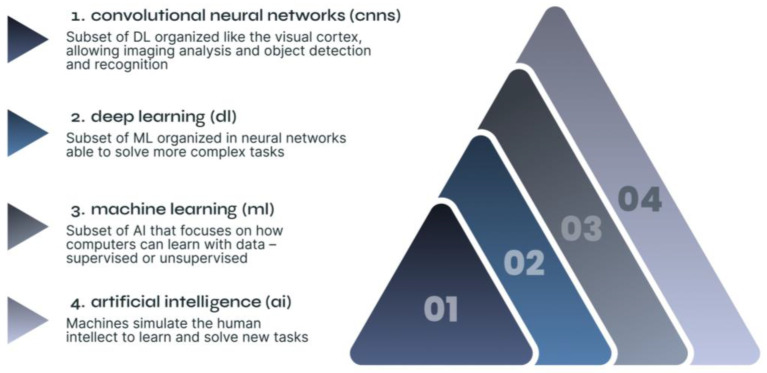
AI’s relationships.

DL, a subset of ML, is organized in neural networks with multiple layers to perform more complex tasks [22]. In deep neural networks (DNN), input layers work with elementary information and, as it progresses through its layers, it becomes more detailed until reaching the output layer where important aspects for the input data’s discrimination are amplified [23]. DL uses convolutional neural networks (CNNs), a class of DNN developed to replicate the visual cortex’ structure and organization. Due to their similar organization to neurons’ connectivity pattern, CNNs outperform other types of DL in visual imaging analysis like detecting and recognizing objects [24,25]. Given this, both DL and CNNs have promising potential in urogynecology, allowing imaging recognition, reconstruction, processing, automated analysis, and classification [3,9,26] (Figure 1 and Table 1).

### 2.2. Key AI Techniques Used in Medicine

ML has two categories [27]. Supervised ML focuses on classification and aims to predict a predefined output or risk [6]. To learn new functions, the algorithm uses training data like images from IEs and maps the input’s variables and features onto a qualitative or quantitative output [19,28]. It is important to notice that it depends on high-quality labeled data. Then, its external validity and generalization are evaluated by testing the algorithm in a new patient set to assess its performance [21].

Unsupervised ML identifies patterns or groupings within unlabeled data on its own, having no outputs to predict [6]. It is a data-driven technique that automatically learns based on the relationship between essential pieces of information and each dataset variable. It depends on the arbitrary aggregation of unlabeled datasets and generates groups based on similarities, including ones that might not have been noticed before [28,29].

Both categories can be applied in urogynecology, enabling outcome prediction and new pattern identification within multidimensional datasets [6].

Natural language processing (NLP) is a subfield of AI that understands human language and infers meaning from unstructured data, ideal for analyzing large-scale databases that use free text like electronic health records (EHRs). These allow physicians to write more naturally without concerns about the computer not recognizing the data and enable physicians to identify complications and adverse events based on EHR data [30]. Thus, NLPs are able to search through large databases and predict outcomes.

Soguero-Ruiz et al. used an NLP model with SVM to search over EHRs for words and phrases that could predict and detect anastomosis leakage after colorectal cancer surgery earlier with a sensitivity of 100% and a specificity of 72% [31]. NLP can generate diagnostic models to detect early-stage chronic diseases and can be found in the form of patient-interactive chatbots, answering their questions and providing relevant and personalized information. NLP can be applied to research, clinical coding, diagnostics, patient care, patient-facing interfaces, and classifying IE reports, enabling physicians to assess treatments and interventions’ efficacies and successfully predict hospital admissions in a shorter time [15,32].

Computer Vision (CV), a subset of AI, allows computers to simulate human vision and uses algorithms to conclude and act based on the acquired visual data [33]. CV was initially combined with ML to handle more data; however, DL has gained an important role with CV, enhancing its performance and presenting great results for complex analysis problems and visual data analysis [34]. Current research centers on teaching computers surgical steps and tool identification/tracking. CV combined with CNNs can identify surgical instruments covered in blood, in different positions, or in blurry images, and analyze surgical videos. Additionally, CNNs and LSTM neural networks enable computers to understand a time point in the procedure [35].

Considering that, CV can harvest useful information from digital images and use it in segmentation, object recognition, reconstruction and detection, earlier detection and characterization of diseases, better selecting patients for early interventions, and better defining treatment and follow-up [34].

### 2.3. AI in Medical Imaging

Humans operate most imaging systems, not being exempt from making errors due to experience level, stress, work overload, or lack of sleep. AI can have a beneficial impact on this domain by improving imaging interpretation, reducing physicians’ workload and the chances of details being overlooked [5,36].

Urogynecology evaluates stress urinary incontinence (SUI) and POP with ultrasounds since they are inexpensive, radiation-free, and allow dynamic evaluations. However, they are highly dependent on the sonographer’s skills, the physician’s interpretation capacity, the scanner, and the patient [37,38]. DL and CNN computer-aided systems improve these limitations and ameliorate the detection, diagnosis, classification, and segmentation of ultrasound images. These systems identify anatomic structures and lesions, classify diseases into categories, evaluate disease status, delineate lesion boundaries, register images, and retrieve content [38].

MRIs and CT scans are widely used in urogynecology. MRIs show greater anatomic details for soft tissues, but image acquisition takes a long time, while CT scans are faster but not radiation-free. Implementing DL tools like CNNs for these imaging modalities enables the improvement of data acquisition, image quality, and registration, decreases segmentation time, detects lesions that could have been missed, accurately quantifies features, and reduces physicians’ workflow [39,40].

## 3. Chapter 2: AI in Diagnosis and Screening

### 3.1. Clinical Evaluation and AI

AI can help in accurately diagnosing PFDs and reducing chances of misdiagnosis by analyzing complex datasets like EHRs, anamneses, and IE, and using unsupervised ML to detect patterns that might match the diagnosis of PFD [8].

AI tools can help physicians during anamnesis as chatbots can access EHRs and conduct more oriented anamneses according to known health conditions and symptoms mentioned, without overlooking any important information. Afterward, AI can integrate all the collected information into EHRs, offering differential diagnoses that require professional evaluation and validation [41]. In this context, NLP can identify keywords in the anamnesis that can alert to disease risks [30].

### 3.2. Imaging and Diagnostic Techniques

Urodynamics tests (UTs) face challenges like characterizing normal parameters in middle-aged and older women. Additionally, their interpretation is not exempt from errors as sometimes their findings do not match the symptoms reported. AI can automate manual analyses and inputs in urodynamics hence avoiding expensive costs, and excessive time spent in analyzing results and reducing human error chances [15]. Wang et al. developed an ML predictive model with manifold learning and dynamic time warping for UTs with an accuracy of 81.35%, a sensitivity of 76.92%, and a specificity of 81.41% to detect detrusor overactivity events, allowing for standardization and more reliable UTs interpretation [16,42]. Similarly, Hobbs et al. created an ML algorithm with SVM that interprets UTs accurately and identifies detrusor overactivity in spina bifida patients with an AUC of 91.9%, the sensitivity of 84.2%, and specificity of 86.4% for their time-based model with 3 channels and an AUC of 90.5%, the sensitivity of 68.3% and specificity of 92.9% for their 3-channel frequency-based model [43].

AI can aid in analyzing MRIs and CTs as seen by Onal et al. where they reviewed 15 MRIs in midsagittal view using five pelvic floor measurement reference points that were to be identified manually and with their semiautomated pelvic floor measurement model. Their model with SVM and k-means clustering presented highly consistent and accurate locations for all points of reference and at a faster speed, showing its applicability to facilitate and improve pelvic floor measurements on MRIs. Additionally, it could improve the correlation analysis with clinical outcomes, improving POP assessment. Nekooeimehr et al. developed a CSHMM that automatically tracks and segments pelvic organs on dynamic MRIs with a Dice similarity index > 78% and classifies multiple-object trajectory, helping to better comprehend POP. It can quantitatively analyze pelvic organs’ movement on MRI, complement clinical examination, and improve treatment and outcomes. Furthermore, it can automatically track, segment, and classify structures from an imaging sequence [16,44,45]. Wang et al. created a ResNet-50 multi-label classification model that simultaneously diagnoses three types of prolapse based on MRI images in 0.18 s with an AUC of 91% and average precision of 84% [46].

SUI diagnosis remains complicated as invasive exams like UTs are only conducted before surgery in complicated patients while less invasive clinical evaluations and questionnaires are time-consuming and sometimes redundant. To modify this panorama, Zhang et al. developed a CNN algorithm based on an Inception-V3 model with transfer learning, using 2D transperineal ultrasound static images to simplify SUI’s diagnostic process, demonstrating an AUC of 92.2% and accuracy of 86.3% [47].

Szentimrey et al. created a nnU-Net segmentation model that could sharpen the efficiency of transperineal ultrasound by reducing the time needed for analysis (1.27 s vs. 15 min) and that had high reproducibility, presenting significant Dice similarity coefficients for the bladder (87.4%), the rectum (68.5%) and the anorectal angle (49.2%) [48,49]. Yin and Wang created an effective CNN algorithm to enhance ultrasonic images’ processing, allowing them to measure the effect of pelvic floor rehabilitation training in pregnant women with POP with a sensitivity of 93%, a positive predictive value of 98%, and a Dice coefficient of 81% [49,50].

Finally, endoscopy is an imaging modality found in urogynecology. Mascarenhas et al. created the first AI model in the world, a CNN using a ResNet model, for the vaginal area that differentiates between low and high-grade squamous intraepithelial lesions with a sensitivity of 98.7%, specificity of 99.1%, and an accuracy of 98.9%, enhancing colposcopies and boosting the detection of cancerous lesions [7]. Similarly, studies in urology with great initial results created CNNs, including TUMNet and Xception-based models, to identify tumors in cystoscopy videos and images [51]. Besides its clinical application in cervical and urothelial cancers, AI can also be used in ovarian cancer as seen in a study by Aramendía-Vidaurreta et al. where they developed a CNN model that combined patients’ age and their ultrasound images’ features to discriminate between benign and malignant adnexal masses with a sensitivity of 98.5%, specificity of 98.9% and global accuracy of 98.8% [3,52].

### 3.3. Predictive Analytics

Throughout the years, AI evolved into complex predictive models [53]. Taylor et al. tested multiple models like RF, XGBoost, AdaBoost, VSM, and Elastic Net, concluding that XGBoost was the best to predict UTIs based on EHRs and variables like vitals, chief complaint and exam results, with an AUC of 90.4%, a sensitivity of 61.7%, a specificity of 94.9% and an accuracy of 87.5%, allowing the early detection of UTIs as urine cultures, the gold standard for diagnosis, take up to 48 h to be available. Thereby, it avoids the unnecessary use of antibiotics, avoids contributing to antibiotic resistance, and allows early treatment in patients with UTIs [54]. Dedeene et al. investigated models like MLP, MLR, RF, XGBoost, and KNNs and used an ensemble voting classifier to combine them, to create an AI model with ML that could rapidly predict the results of urinary culture tests using urinalysis results, sample collection method and patient demographics with AUC-ROC values of >85% [55]. Burton et al. tested RF, XGBoost, and Neural Network models to identify urine samples that would produce negative results to reduce laboratory diagnostic workload, concluding that the XGBoost algorithm was the best one with a sensitivity of >95% [56]. Beyond identifying UTIs, AI tools can identify patients at higher risk of developing sepsis and other preventable complications, alerting doctors earlier [57].

AI is pivotal in oncology, which benefits urogynecology as it encompasses renal and bladder cancer. AI can interpret imaging and histological exams, predict tumor grades, identify genomic biomarkers, and reduce inter-observer variability, enhancing diagnosis precision and allowing physicians to make the best clinical decision [58]. AI can access EHRs and interpret symptoms and signs, which could benefit urogynecology with the aid of NLP, WD, and the integration with IE, enabling earlier cancer detection and decreasing missed diagnoses [59].

## 4. Chapter 3: AI in Treatment and Management

### 4.1. Personalized Treatment Plans

Treatment planning in urogynecology could greatly benefit from AI as it has the capacity to review the extensive medical literature and databases, integrate its findings with the patient’s data, and recommend the most appropriate treatment based on its findings and the patient’s profile [8].

In PFDs, AI can help physiotherapists by providing clinical decision support and data analysis, creating personalized interventions for each patient, and offering remote monitoring through WDs. This way, AI can improve performance and results, engage patients, and educate them [15].

### 4.2. Surgical Interventions

AI can help surgeons with pre-operative planning and with intraoperative guidance. AI and augmented reality (AR) can make a significant impact in both surgical planning and training as surgeons wear a headset that superimposes their real-world vision with digital images, not isolating them from the world [53]. CV is also of great importance for analyzing and interpreting visual data using DL and CNNs, enabling better decision-making processes, safer surgeries, and better outcomes [60].

AR with CV can help surgeons to perform precise and intrinsic movements during the operation. It provides real-time feedback throughout the surgery like alerts to prevent risks or errors [8,60]. Moreover, it offers data analysis, decision support, and precision control, enhancing the surgeon’s performance and allowing them to learn from previous cases [15,60]. ML can predict pre- and post-operative complications, blood loss, post-operative mortality, pain, and more. Furthermore, ML and CV can recognize critical surgery steps in videos and warn surgeons of deviations and omissions of critical steps, reducing errors and offering better guidance [60,61]. Additionally, the superimposition of previous IEs onto the operative field enables better guidance, identification of anatomical structures, and avoidance of complications by highlighting fragile structures [53].

CV has many uses in the operating room (OR): assessing a case’s difficulty, warning surgeons against incising in the wrong place, guiding safer dissections, and improving OR communication and OR staff awareness [60].

The ureter is prone to injuries during surgeries, possibly leading to fistulas and even loss of renal function [62]. This happens because during laparoscopies surgeons cannot rely on tactile senses, only on visual information. With that in mind, Serban et al. created ensembles of binary semantic segmentation networks to distinguish and detect the uterine artery, the ureter, and nerves during laparoscopy. Their binary U-Net model for the ureter presented a Jaccard score of 81.92% and a Dice score of 89.28% [63]. Narihiro et al. developed a DL-based semantic segmentation algorithm called UreterNet to recognize the ureter with a precision of 71.2%, a recall of 72.2%, and a Dice coefficient of 71.6%; however, it is necessary to assess if this model reduces iatrogenic ureteral injuries [64]. Additionally, Kitaguchi et al. developed real-time automatic ureter and autonomic nerve recognition models for colorectal laparoscopic surgeries: UreterNet and NerveNet, both DL-based CV models. Their success rate was ≥89% in correlation with the surgeon and could recognize structures faster than surgeons 75% of the time. Their ureter recognition rate was 95%; however, this study presents several limitations, needing further studies such as randomized controlled trials and multicentric studies to demonstrate its clinical benefits [65]. Chen et al. created three models with ML using neural networks, RF, and XGB to predict the risk of ureter injury during colorectal surgery. Their XGB model presented the best results with an AUROC score of 77.4% (95% CI 0.742–0.807) helping surgeons decide if the patient could benefit or not from a ureter stent [66]. Yu et al. designed an image-guided endoscope system able to capture coeliac imaging and detect the ureteral position with a true positive rate of 93.8% and a positive predictive value of 90.6%, making it possible to decrease iatrogenic ureter injury; however, this model is yet to be tested on humans [67].

One of pelvic floor surgery’s main challenges is to comprehend the pelvic anatomy from a three-dimensional point of view, including musculature relationships, ligamentous supports, and nearby neurovascular structures. Learning these concepts is hard, which is why AI could have an important role in this domain. Siff et al. created an interactive holographic curriculum with an AR headset for uterosacral ligament suspension and sacrospinous ligament fixation, aiming to teach trainees anatomy, procedural steps, and recognition and management of these. A total of 88% of trainees found the training with AR “much” or “very much better” than the usual self-study, while 81% were “likely” or “very much likely” to use AR to prepare for surgery [68].

AR is very present in robotic surgery, being able to be applied in minimally invasive procedures. In urogynecology, it can be used in robot-assisted sacrocolpopexy, hysterectomy, or sling placement, identifying the mesh anchorage points’ correct placement and highlighting blood vessels, ureters, and other structures. Its advantages are the decrease in many procedures’ learning curves, human error, and operative times, enhancing safety and outcomes [15]. This way, AI can have a big impact on urogynecological surgeries, especially on those who use fiber optic cameras like hysteroscopies and laparoscopies.

### 4.3. Pharmacological Management

Precision medicine predicts which treatment protocol will have the best results on a patient based on their characteristics. This is achieved by analyzing the patient’s data, genetics, lifestyle, and environmental factors via unsupervised ML, which finds undiscovered relationships and patterns in their data, assesses their condition, and recommends the most appropriate treatment in a cost-effective manner [4,6,14,69].

UI is managed with anticholinergics and beta-agonists. AI can play a big role in drug selection according to the patient’s characteristics, symptoms, and treatment steps to select the most adequate drug. AI can analyze drug databases to anticipate their therapeutic potential and favor target prioritization. By doing this, AI also considers drug interactions with the patient’s medication and drug-target interactions. Antimuscarinics like Oxybutynin are prescribed for urge incontinence and some patients develop side effects. In this case, AI can help select a better drug for them and reduce the number of urogynecology appointments a patient might have just to adjust their medication. Therefore, it is possible for AI to optimize treatment protocols [15].

Sheyn et al. developed an ML prediction model with RF for anticholinergic response in patients with overactive bladder (OAB) with a sensibility of 80.4% and specificity of 77.4%. This model allows patients likely to benefit from this treatment to receive it, sparing others from unwanted side effects from the medication [16,70].

## 5. Chapter 4: AI in Patient Monitoring and Follow-Up

### 5.1. Remote Monitoring Tools

Remote monitoring tools like WDs with AI allow continuous virtual monitoring, tracking, and management of a patient’s condition, allowing physicians to remotely access their collected data [71]. They also empower patients to collect their own health data, allowing quick medical interventions if needed [4]. This makes healthcare more accessible for patients who live far away and for those with limited mobility. When it comes to urogynecology, WDs can be used for UI monitoring and to monitor post-void residual bladder volume scanners [71].

Kuru et al. created a WD to monitor the bladder with ultrasounds combined with ML, with a sensitivity of 89% and a specificity of 93% to determine imminent voiding need in children with nocturnal enuresis. This system’s data were analyzed with three ML techniques: SMO, LR, and EB, and the bladder volume data acquired from the ultrasound with a pre-trained model, being able to process the volume and trigger an alarm to warn patients that their bladder is almost full. ML training models enable personalization by considering different variables like sex, age, and bladder morphology, which increases its accuracy [72,73].

UI is often tracked by voiding diaries, which face challenges like children and the elderly not being able to record all variables, leading to inaccuracies and varying levels of compliance. Kim et al. created a system using RNN and CNN to replicate the voiding diary’s function, with an average accuracy of 94.2% in recognizing urinary tract activity and 83% for preventing neurogenic bladder with a motion-analysis technology. This system, integrated with smartphones and WDs, collects signal information like visual and motion data and enables automatic recording. This way, by analyzing data from a smart band, they enabled AI to monitor urination [74]. Similarly, Eun et al. developed an RNN-based LSTM method with WDs that recognizes urination time and spacing based on the patient’s posture and its changes with 95.8% accuracy [75].

Many health apps are available for urogynecology; however, only a few are accurate concerning information and clinical decision-making [76]. An example is the app Tät^®^ for pelvic floor muscle training, which, according to Nyström et al. has short and long-term effects on the patient’s symptoms and quality of life (QoL). This way, the self-management of UI is possible through an app, can reach larger demographics, and includes features like lifestyle advice, reminders, and graphics [77]. Another of their use is to educate patients, as seen by Han et al. with the app Bwom© version 2.5.8, which educates patients about pelvic floor exercises with personalized plans based on risk factors [78]. Overall, health apps allow patients to track symptoms and medication adherence, create personalized treatment plans, and empower patients to monitor their health [79].

### 5.2. Telemedicine and AI

By having a patient’s EHR, software can automate their schedules, care, and treatment plans, and alert them for follow-ups. Besides that, WDs can be applied for remote monitoring. Thus, virtual consultations in urogynecology could benefit from AI as physicians would be able to evaluate patients who live far away or who have restricted mobility and would have all the data needed to be collected from WDs, without troubling the patient and reducing wait times [16].

Additionally, AI systems can be available for patients, helping them to find available physicians in their area, scheduling appointments, and answering questions. Doctors can access such systems through mobile apps to search for protocols, available clinical tools, and drugs in the hospital [4].

Lucas et al. revealed that post-traumatic stress disorder patients were more likely to talk to chatbots than to humans due to fear of judgment, resulting in honest answers from patients, which allowed doctors to better diagnose and treat them [4,80]. This is important in urogynecology because UI and OAB are still considered taboo. An Austrian female population study revealed that 60.6% considered UI as taboo, leading to isolation, reduction in QoL, and low rates of consultation and treatment [81]. A recent OAB population study showed that women felt embarrassed, invalidated, and dismissed by physicians because of OAB [82]. Thus, AI could help elevate urogynecology consultation rates for these conditions through chatbots that allow patients to express their concerns, describe their symptoms, and provide them with personalized information and options for scheduling consultations.

### 5.3. Predictive Maintenance of Health

POP surgery is not exempt from complications. Sometimes, pelvic anatomy reconstruction surgery might not improve women’s QoL, highlighting the importance of predicting which patients will benefit from it and which ones from targeted therapies. By including QoL scales and symptoms in AI systems, POP patients might benefit from a more accurate and effective analysis of their diagnosis and follow-up after treatment, leading to better management of PFD [16].

Trebeschi et al. created a PAM model that predicts the prognosis of metastatic urothelial cancer in patients receiving immunotherapy. This model assesses follow-up chest and abdominal CTs for tumor morphological changes, spread, and side effects, and links them to the patient’s 1-year survival, reaching an AUC of 67% for chest and 73% for abdominal CTs [83]. These applications enable urogynecologists to better predict treatment outcomes, prevent complications better comprehend patients’ conditions, and provide a clinical decision support system.

## 6. Chapter 5: Quality of Life and Psychosocial Impact

### 6.1. Improving Patient Outcomes

Patients’ safety might be compromised due to drug adverse side effects (DASEs), surgical complications, infections, decompensation, and diagnostic errors. AI can identify patients at risk through their EHR, vitals, and data collected by sensors, test results, and IEs enabling early prevention, and improving diagnostic accuracy and infection control. DASEs can be predicted using DL, identifying the molecular substances at cause and offering personalized treatment options and risk estimations. Regarding decompensation, with WDs, AI can recognize its early signs, avoiding life-threatening complications like sepsis. Finally, AI can reduce diagnostic errors by helping with classifications, imaging interpretation, and enabling early detection [61].

AI can be applied to predict which medication patients are taking but are not in their EHRs and to monitor medication intake [84]. With those applications, AI can have a positive impact on QoL. However, these algorithms have not been validated externally yet and come from retrospective studies, so they might be biased, and their generalization might be limited [61].

### 6.2. Ethical and Psychosocial Considerations

“AI anxiety” refers to the fear of AI’s quick integration into our daily lives, creating doubts about job security, privacy, biases in data, and more [85]. Claims that AI will replace physicians in the future are commonly heard but this is not what AI is intended for, and neither is a possible scenario as doctors are fundamental for the human part of medicine and needed to critically assess AI tools’ decisions. AI should be viewed as a complementary tool that can be integrated into clinical practice and help clinicians by freeing their time to make room for more patients, helping them notice new unexplored patterns, and offering support for interpretations and decision-making [86].

A study by Wang et al. aimed to outline patients’ perception of AI being integrated into healthcare systems, revealing optimism in patients for the adoption of AI in healthcare, with 70% agreeing or strongly agreeing that AI in healthcare will be a general trend in the future. However, over 60% of patients presented concerns over AI being misused due to security risks, lack of knowledge, and regulation flaws, possibly leading to negative outcomes [87]. Similarly, a study by Tran et al. aimed to understand how patients perceived AI and WDs in healthcare in a French population with chronic conditions. A total of 20% of patients considered that the benefits of AI outweighed the dangers while only 3% considered the opposite. A total of 47% considered AI and WDs a great opportunity and identified 47 benefits of their use such as improvement in follow-up and reactivity of care (55%), reduction in their treatment burden (23%), and helping physicians’ work (21%). On the other hand, 11% considered AI and WDs a great danger, identifying 31 potential risks such as fear of replacing human intelligence in care (28%), misuse of private information (14%), and hacking (13%). Finally, 13% of patients were globally against any use of AI or WDs in their care while 22% would refuse AI and WDs in one presented scenario and 65% would agree with their integration in their care in all presented scenarios [88].

These tools require extensive amounts of data to be trained, and this might put patients’ privacy at risk since anonymization and re-identification are time-consuming and hard to execute [3]. Safeguarding medical data is becoming increasingly challenging due to cyberattacks, data vulnerability to manipulation and remote access, and the fact that electronic data are easily reproduced. Blockchain technologies offer a solution to these concerns, providing safer and traceable handling and storage of data, assuring its immutability. However, patients are required to consent to their data being used to train these tools and can choose what they want to share [4,86].

## 7. Chapter 6: AI in Research and Development

### 7.1. Accelerating Research

Concerning big data, databases like urogynecological care cloud platforms can receive, record, and analyze patients’ data and match them with diseases in the database. WDs and hospital systems’ data are uploaded into the cloud and AI allows the automated integration of EHR with images and other data, improving decision support [36,89].

Such databases are important for data storage and backup and use data mining, AI, and other techniques to improve treatment options for conditions like PFDs, improving rehabilitation and patient management by giving physicians feedback from urogynecological centers and offering suggestions. This platform improves data-sharing by promoting interactions with other medical departments and urogynecology centers and enables patients to access their data anytime [89].

### 7.2. Clinical Trials and AI

Doctors are aware of clinical trial opportunities, but most do not have the time to go through each patient’s EHR to evaluate which trial is ideal for them. A total of 20% of clinical trials fail to complete enrollment and >40% are terminated due to low enrollment [90]. AI can help in patient recruitment using ML and NLP in datasets like EHRs and medical literature to better select and match patients to the most suitable trial, leading to higher recruitment rates in less time, better compliance control, and more effective and reliable endpoint evaluations. Additionally, AI allows patients’ data monitoring and analysis, improving measurements and results interpretation [91]. This way, AI in clinical trials can improve their efficiency, and reduce costs and their likelihood of failure by recruiting suitable patients [90,92].

AI can predict potential dropouts based on the participant’s data, enabling a timely intervention by physicians to try to retain them [91,93]. Conversely, it can predict which patients might reach endpoints earlier, shortening trial durations [91].

### 7.3. Innovative Research Methodologies

AI quickly processes similar studies, clinical data, and regulatory information and interprets relevant data, processing big data in a shorter time and detecting new data patterns, giving its integration with traditional research approaches an advantage [92,94]. This way, AI introduction in research can help with trial design, implementation, and analysis, increase the identification and management of risks, and the research’s efficiency due to its automated nature and predictive power via ML and NLP [93].

**Figure 2:**
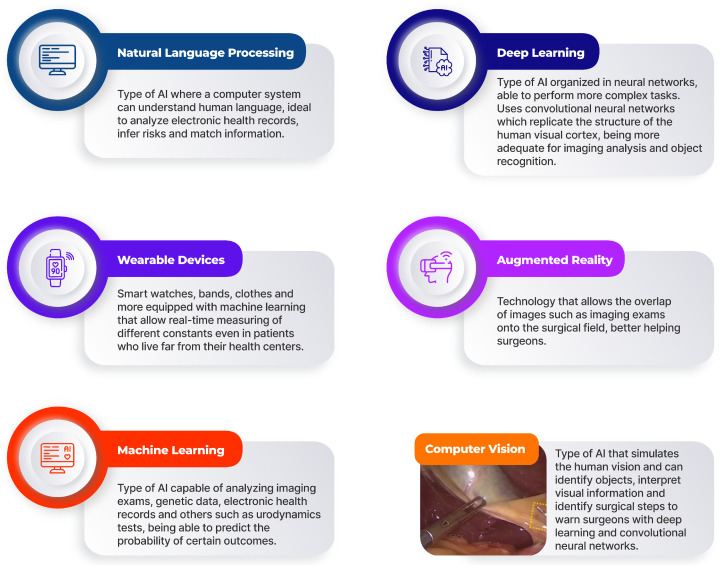
Different AI types and their applications in medicine. Image from the Computer Vision section taken from [60].

With healthcare data’s large volume, AI allows for a more accurate data-driven hypothesis generation, analyzing the scientific literature, detecting insights and similarities between datasets, and generating hypotheses from that [95,96]. These tools use resources more efficiently as they focus on a targeted area of interest for a specific hypothesis, allowing meaningful insights and more interpretable and explainable results. Additionally, these tools also test and validate those hypotheses with AI [97]. This way, urogynecology can benefit as AI would be able to hypothesize about tumor stages in cancer, possible risk factors of urogynecological conditions, possible surgery and procedure complications, and more, all based on the patient’s clinical data (Figure 2).

## 8. Chapter 7: Challenges and Limitations

### 8.1. Technical Challenges

Besides requiring large data availability and its privacy implications, AI also faces challenges like bias introduction during data training and tool designing, which compromises algorithmic fairness, and spectrum bias, which occurs when a diagnostic test is run in a different population, highlighting the importance of assessing if AI models are valid and applicable to a new dataset before implementing them, and of assessing the models’ overfitting. These can be avoided by training models with larger datasets. Hence, programs that ensure ethical AI development are required to prevent biases [86]. It is also important to watch for biased data, preprocess collected data, select the best algorithm for a task, and use the appropriate metrics for a problem [4].

AI tools might not perform well in a different environment, meaning their transferability is not always guaranteed, possibly resulting in fragmented data between institutions. Even if they are retrained for the new environment, they might not perform as well, making it vital for each tool to be carefully designed, tested, and evaluated before its implementation, and to be transparent about the data sources used in their design and development and the data safety demands [4,86]. Furthermore, the digitalization of medical records and data standardization by healthcare institutions is needed in order to enable AI’s implementation [4].

It is important to make models more accessible and interpretable to ensure AI tools’ explainability to avoid black-box medicine and the break of trust in doctor-patient relationships. Additionally, it is important to address the responsibility and accountability regarding AI tools’ decisions and adverse events that might arise from their use, as it could fall into the hands of the physician, healthcare institution, or tool designer and can influence the patients’ trust [4,86].

### 8.2. Clinical Integration

With the increasing medical knowledge available, AI is needed to enable physicians to apply this knowledge to their clinical practice [98]. However, many healthcare workers do not have AI literacy, which hinders its implementation [99]. Consequently, it is important to promote AI education in their formation, teaching the fundamentals of AI to benefit the health system and create safe algorithms since one bad one can harm multiple patients [100,101]. This way, it is important that healthcare workers have comprehensive training and know how to create a bias-free algorithm, use and critically analyze it, and know about the ethical and legal aspects of AI [100,101,102]. Furthermore, physicians need to train emotional intelligence and communication skills since AI lacks those when interacting with patients. Finally, AI brings many advantages that must be taught like cost reduction, better quality, and healthcare access, but on the other hand, it is equally important to know about its pitfalls like transparency and liability [98]. To guarantee that algorithms are validated and reliable, it is important to have experts from various fields like medicine, data science, law, ethics, and engineering, to fight AI illiteracy and promote its safe implementation [8,100,101].

Integration with existing healthcare systems is very important as the information must be shared across institutions and their software quickly and efficiently, making sure the AI system can interoperate with the hospital’s system [103,104]. However, AI’s implementation in healthcare is limited due to the continuous need to validate and test algorithms to check their sensitivity and specificity and the need to ensure unbiased data, generalization, and representative results. Moreover, it is necessary to ensure integration with clinical workflow since models that are too complex or require additional work are less likely to be implemented [104].

### 8.3. Regulatory and Ethical Issues

Regarding regulatory frameworks, the European Union (EU) signed the AI Act in 2024 to guarantee these systems are transparent, safe, traceable, non-discriminatory, and environmentally friendly. Healthcare-related AI is classified as high-risk, so before being applied, it must comply with EU requirements on risk management, testing, data training and governance, technical robustness, transparency, cybersecurity, and human oversight [105]. Due to AI’s disruptive native, new regulatory frameworks and guidelines are expected to arise, being important to address the privacy of patients, data security and bias, explainability, transferability, and responsibility before implementing an AI tool [8,86].

**Figure 3:**
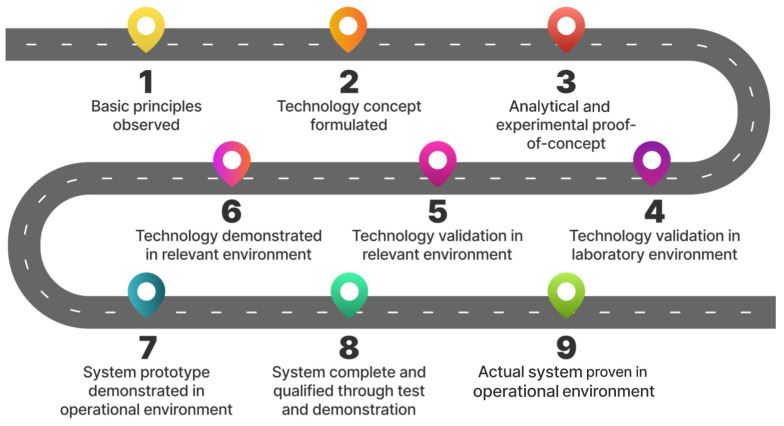
Technology readiness level scale.

NASA has created the technology readiness level (TRL) scale which evaluates a technology’s maturity level, ranging from 1 to 9 [106]. Currently, most AI tools in urogynecology are at TRL5 and 6 as they are slowly tested in relevant settings. However, there is still a long way to go, and more research is needed for them to reach TRL9 and be implemented (Figure 3).

## 9. Chapter 8: Case Studies and Real-World Applications

### Successful Implementations

AI in urogynecology has a huge potential and many thrilling opportunities awaiting. As seen in this review, AI has been applied in urogynecology in IEs, UTs, surgery, telemedicine, consultations, and research, highlighting its versatile potential for urogynecology and the advantages it can bring to patients and healthcare workers. With these applications in mind, AI in urogynecology still has challenges to face before its clinical implementation but has the potential to deliver patient-centered solutions [71].

For better future AI outcomes in urogynecology, it is important to note that most studies conducted are retrospective and there are not enough prospective studies that validate AI in clinical environments [107]. Additionally, it is important to address concerns over AI training methodology and datasets, the biases they might carry, and the algorithmic complexity that hinders its interpretation for physicians and patients [58]. As new knowledge is gained, new types of errors might arise and it is important to always respect ethical aspects and the patient’s privacy, highlighting the importance of developing regulatory approval guidelines for AI tools aiming to protect patients and physicians [71]. Finally, it is important that AI algorithms are developed by a multidisciplinary team to ensure their best functioning.

## 10. Conclusions

### 10.1. Summary of Key Findings

AI will greatly benefit urogynecology in the next few years. Physicians will use AI to better take anamneses, predict health risks and outcomes, better choose medication, interpret IEs and UTs, better prepare surgical procedures, monitor patients, communicate with patients, better refer patients, and better conduct clinical trials and research.

Patients always seek the best care and AI enables personalized medicine, allowing doctors to find the best treatment and management plans for patients based on their medical history, genetics, age, lifestyle, family history, laboratory and IEs, and medication. Most urogynecology patients are older women who have many different conditions at the same time and AI can help to detect them early, even when they might be difficult to detect. Telemedicine’s rise with WDs and health apps also provides physicians with higher-quality data from patients, which can improve their care experience.

Patient benefits include timely prevention of diseases and complications, more accurate and earlier diagnoses, safer procedures, reduction in DASEs and diagnostic errors, bigger access to healthcare, shorter wait times, better monitoring and follow-up, and better trial-matching. As for urogynecologists, AI reduces the workload and time spent analyzing and interpreting EHRs and exams, freeing more time in their agendas that can be dedicated to seeing more patients. It can help with better decision-making processes by detecting unseen patterns and risk factors, integrating real-time data into the patient’s EHR, predicting risks and outcomes, improving communication with patients, and offering guidance during surgeries. As for interns, AI can enhance their surgical education with surgical guidance and performance evaluation.

### 10.2. Concluding Remarks

AI in urogynecology has only begun but is quickly maturing and pushing in many directions. Due to its many advantages, AI is currently in the medical field’s spotlight and can potentially play a central role in women’s pelvic floor health in the future. There are increasing advancements being made in this field and many exciting developments are expected to happen; however, there is still a long way to go and many barriers to be overcome before AI’s clinical implementation. Urogynecologists will have to learn how to operate AI systems and more research studies addressing AI’s limitations and how to overcome them are needed. Changes in the healthcare industry like the digitalization of EHRs and standardization of data are also fundamental. Without this cooperation, AI’s implementation in the clinical setting will remain a challenge.

## Figures and Tables

**Table 1:** Machine learning (ML) and deep learning (DL) models discussed in this review and their definitions.

Type of Model	Model	Definition
ML	Support Vector Machine (SVM)	Supervised algorithm used for classification and regression tasks, searching the ideal hyperplane to categorize data in a high-dimensional space while maximizing the margin between them.
	Random Forest (RF)	Algorithm used for classification and regression tasks that generates multiple decision trees, yielding the categories’ mode or the mean prediction, respectively, mitigating overfitting in the training set.
	Extreme Gradient Boosting (XGBoost)	Gradient boosting algorithm used for classification and regression tasks which combines sequentially multiple decision trees’ predictions originating more precise and robust models.
	k-Nearest Neighbors (kNNs)	Algorithm used for classification and regression tasks which uses a non-parametric supervised method to analyze the most common class or the average of the k value closest to the data points to make predictions.
	K-Means Clustering	Non-supervised algorithm used to cluster data to separate it into k different groups based on shared similarities.
	Coupled Switched Hidden Markov Model (CSHMM)	Algorithm used to analyze complex data such as time series from various intertwined processes and fluid data interactions.
	Manifold Learning	Method to simplify and better comprehend multidimensional complex data to facilitate working with them.
	Adaptive Boosting (AdaBoost)	Algorithm used for classification tasks that combines multiple weak classifiers to originate a stronger one, adjusting the weight of the training data so that errors would receive more attention.
	Elastic Net	Regression method used for datasets with many predictor variables, able to improve the model’s stability and the selection of variables by combining Lasso (L1) and Ridge (L2) penalties.
	Multilayer Perceptron (MLP)	Artificial neural network used for classification and regression tasks where each neuron is connected to all neurons in the following layer, using a non-linear activation function to facilitate teaching complex data patterns.
	Multiple Logistic Regression (MLR)	Supervised algorithm used for classification tasks based on several independent variables, enabling effective modeling and the prediction of categorical outcomes.
	Ensemble Voting Classifier	Technique that gathers and combines different models to make enhanced predictions based on the different models’ outputs.
	Neural Network	Model inspired by the human brain that is able to identify patterns and make predictions due to its interconnected neural layers.
	Sequential Minimal Optimization (SMO)	Training algorithm for SVMs that aims to solve its optimization problem by finding the ideal hyperplane for separating different data classes.
	Linear Regression (LR)	ML algorithm used for regression tasks, able to make predictions and discover data trends.
	Ensemble Bagging (EB)	ML technique that generates many training dataset subsets and uses each to train a model, originating predictions for each subset that are then combined to create a final prediction, reducing overfitting and variance.
	Prognostic AI-Monitor (PAM)	Algorithm that predicts healthcare outcomes, risks, and events based on healthcare data analysis.
DL	Convolutional Neural Networks (CNNs)	Neural network which processes multidimensional grid-topology data like images, enabling image classification, object recognition and detection, segmentation, and more, being highly used in computer vision.
	U-NET	Type of CNN used in image segmentation tasks at the pixel level, being applied in the biomedical domain.
	Residual Network (ResNET)	DL architecture that uses residual connections to improve information flow and facilitate training, all while diminishing the vanishing gradient problem.
	ResNet-50	Popular version of ResNET with 50 layers created to improve deep neural networks’ performance in imaging classification.
	Inception-V3	CNN architecture used for image recognition and identification tasks and for transfer learning, able to analyze different parts of the image at the same time.
	Transfer Learning	Technique where a model created to perform a task is adapted to perform a different task by drawing insights from a pre-trained model.
	No-new U-Net (nnU-Net)	Advanced framework used for medical image segmentation at pixel level which automates part of the required training enabling it to adapt itself to different databases without broad manual training.
	TUMNet	DL architecture used for medical imaging segmentation, more specifically for tumor detection and analysis.
	Xception	Type of CNN used for image classification and segmentation, object detection, image and video analysis, and computer vision tasks.
	Recurrent Neural Networks (RNNs)	Artificial neural networks used for processing sequential data, having connections that enable maintaining the memory of past inputs due to them looping back on themselves.
	Long short-term memory (LSTM)	Type of RNN used for sequential data and to address RNNs’ limitations of maintaining long-term data.

## Data Availability

The authors will provide additional information on their research. For more information, please contact the corresponding author.
